# A focus on unexpected surprises in RiPP natural product biosynthesis

**DOI:** 10.1039/d5sc90158h

**Published:** 2025-09-03

**Authors:** Christopher J. Thibodeaux

**Affiliations:** a McGill University, Department of Chemistry 801 Sherbrooke St West Montreal Quebec H3A0B8 Canada christopher.thibodeaux@mcgill.ca

## Abstract

Natural products are biologically active molecules made by living organisms that serve a vital role in the pharmaceutical industry and account for (or have inspired) nearly 75% of human medicines. For decades, natural product biosynthetic enzymes have challenged chemists and enzymologists to harness these powerful catalysts for the production and engineering of high-value, structurally-complex chemicals. As genome science has rapidly advanced over the past two decades, the ribosomally-synthesized and post-translationally modified peptide (RiPP) family of natural products have emerged as a promising target for detailed investigation. Recently, Zhang and co-workers (Y. Jia, Y. Han, X. Liu and Q. Zhang, *Chem. Sci.*, 2025, **16**, 10722, https://doi.org/10.1039/D5SC01546D) reported the characterization of thuricin CD (an antimicrobial RiPP) and revealed several unexpected surprises that have expanded our understanding of natural diversity in RiPP biosynthetic mechanisms. Their study calls for caution when making assumptions about these highly versatile biosynthetic pathways and highlights a need for detailed characterization of these pathways as a prelude to engineering applications.

## Abstract 2

Careful examination of the enzymes involved in the biosynthesis of ribosomally-synthesized and post-translationally modified peptide (RiPP) natural products reveals unexpected synergy and surprising catalytic and biophysical properties.

## Graphical abstract

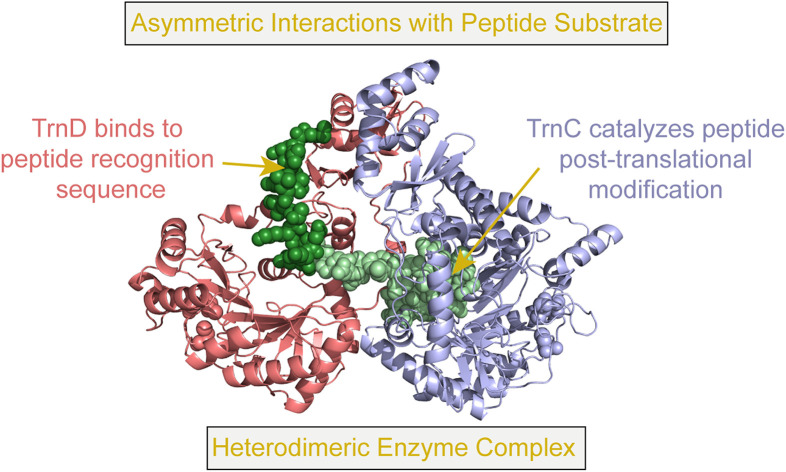

RiPPs are structurally diverse peptide natural products made by all domains of life that possess diverse biological activities.^1,2^ Several features of RiPPs render them ideal targets for manipulation. First, all RiPPs are derived from genetically encoded peptides and are chemically modified by biosynthetic enzymes that often act iteratively on the precursor peptide to install multiple modifications (Fig. 1A). The genetic encodability of the substrate (by a single gene), combined with the relaxed substrate specificity of the biosynthetic enzymes make these systems highly amenable to manipulation by modern biomolecular engineering strategies.^2–6^ Moreover, many RiPPs possess enzymatically-installed peptide macrocycles as the core component of their structure and bioactivity. Macrocycles are important pharmacophores that provide chemical stability and target specificity to biologically active peptides.^7^ RiPP cyclases typically install these macrocycles with both regio- and stereoselectivity to retain the biological activity of the final product. Such control over peptide macrocyclization remains challenging with chemical synthesis and has inspired many efforts to understand the molecular basis of cyclization fidelity in RiPP biosynthesis.

**Fig. 1:**
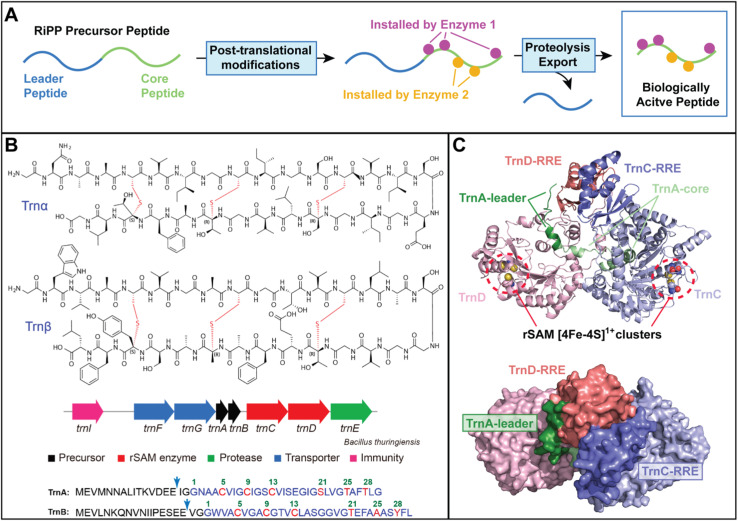
(A) General biosynthetic scheme for RiPP natural products, where biosynthetic enzymes with relaxed substrate specificity install modifications into the genetically encoded precursor peptide. (B) The chemical structures of the post-translationally modified TrnA and TrnB precursor peptides (Trnα and Trnβ, respectively) are shown at the top. The thioether moieties installed by the TrnCD complex are highlighted in red. The thuricin CD biosynthetic gene cluster and complete amino acid sequences of the TrnA and TrnB precursor peptides are shown at the bottom. Reproduced from ref. 11 with permission from the Royal Society of Chemistry, copyright 2025. (C) Alpha Fold 3 models of the TrnACD complex. The structure at the bottom is rotated towards the reader 90° to illustrate the tight interaction between the RiPP recognition elements (RREs) of TrnC and TrnD. In the AF3 model, the TrnA leader peptide interacts primarily with TrnD, while the TrnA core peptide binds into the catalytic core of TrnC. The approximate location of the rSAM [4Fe–4S]^1+^ clusters bound by TrnC and TrnD are indicated.

Ideally, the structures of RiPPs (including macrocycle topology) and the functional properties of RiPP biosynthetic enzymes would be predictable from gene sequence information – allowing researchers to prioritize uncharacterized systems with the desired properties for detailed studies. However, the highly dynamic nature of RiPP precursor peptides (which are typically intrinsically disordered), combined with the structural plasticity of enzyme–peptide binding interactions and the relaxed substrate specificity of RiPP biosynthetic enzymes, has made predicting reaction outcomes in RiPP biosynthetic pathways difficult.^8,9^ In many cases, the functional versatility of RiPP biosynthetic enzymes is likely linked to the intrinsic structural dynamics of RiPPs and of RiPP–enzyme interactions, which can govern the kinetics and sequence of post-translational modification events.^10^ Thus, structure–function relationships in RiPP biosynthesis remain challenging to computationally predict and necessitate detailed investigation of individual systems.

The challenges in predicting biophysical interactions in RiPP pathways sometimes result in surprising discoveries, as highlighted by Zhang and co-workers in their recent work on the two-component sactipeptide antibiotic, thuricin CD (Fig. 1B, https://doi.org/10.1039/D5SC01546D).^11^ Most known RiPPs are derived from biosynthetic gene clusters that encode for a single precursor peptide gene and a single set of biosynthetic enzymes. However, some clusters encode for multiple precursor peptide genes that are modified by the same set of enzymes. Well-characterized examples include the prochlorosins^12^ and certain other class II lanthipeptides,^13^ the cyanobactins,^14^ and the linaridins.^15^ In other “two-component” systems, each RiPP precursor peptide is modified by a dedicated synthetase. Well-characterized examples of two-component RiPPs (which prior to the thuricin CD study by Zhang's team were restricted to lanthipeptides) include haloduracin,^16^ lichenicidin,^17^ and lacticin 3147.^18^

The thuricin CD gene cluster encodes for two precursor peptides (TrnA and TrnB) and two radical *S*-adenosyl methionine (rSAM) enzymes (TrnC and TrnD), along with genes involved in peptide transport, immunity, and proteolysis (Fig. 1B).^19^ Radical SAM enzymes comprise a catalytically versatile superfamily that are widespread in RiPP biosynthetic gene clusters where they often catalyze peptide macrocyclization.^20–22^ Like all rSAM enzymes, TrnC and TrnD utilize a reduced [4Fe–4S]^1+^ cluster to generate a dexoyadenosyl radical (dAdo˙) from SAM.^23,24^ In the case of TrnC/D catalysis, the dAdo˙ species is proposed to trigger the oxidative formation of nested thioether rings in the TrnA/B peptides by linking the sulfur atoms of cysteine residues in the C-terminal part of the TrnA/B core peptides to α-carbons located in the N-terminal region of the core (Fig. 1B).

Initially, using the biosynthetic logic observed in the two-component lanthipeptides, it was assumed that each thuricin CD rSAM enzyme would modify a specific precursor sactipeptide. Surprisingly, using both *in vivo* heterologous expression experiments in *E. coli* and *in vitro* enzymatic assays with purified components, Zhang and colleagues showed that, in isolation, neither TrnC nor TrnD was able to install thioether moieties into either of the thuricin precursor peptides, despite the fact that both enzymes reductively cleaved SAM into 5′-deoxyadenosine (a typical *in vitro* side-reaction catalyzed by active rSAM enzymes in the absence of a substrate). In contrast, complete modification of the TrnA and TrnB peptides was only achieved when both TrnC and TrnD were simultaneously co-incubated with the precursor peptides. Pull-down experiments using His-tagged enzymes, combined with microscale thermophoresis binding assays showed that TrnC/D indeed form a heterodimeric protein complex that binds to either TrnA or TrnB with low micromolar affinity (a typical affinity in RiPP biosynthetic systems).

Delving deeper into this unexpected result, they made a second surprising finding that TrnC and TrnD function asymmetrically within the heterodimer. To accomplish this, they conducted *in vitro* activity assays with TrnC/TrnD heterodimers where one of the two components (either TrnC or TrnD) was replaced with a catalytically inactive variant containing mutations in the conserved cysteine residues that coordinate the catalytically essential rSAM [4Fe–4S]^1+^ cluster. Interestingly, these assays showed that only the [4Fe–4S]^1+^ cluster of TrnC was required for both TrnA and TrnB modification, whereas mutation of the rSAM [4Fe–4S]^1+^ cluster in the TrnD active site had no apparent effect on TrnA/B modification. Subsequent structural modelling using Alpha Fold 3 (AF3) supported this finding and suggested that TrnD plays a more dominant role in binding to the N-terminal leader peptide recognition element of TrnA/B, while the TrnC rSAM active site has greater access to the core peptide modification sites on the C-terminus of the TrnA and TrnB peptides (Fig. 1C).

A final unanticipated observation involved the nature of the putative enzyme–enzyme and enzyme–peptide binding interactions (Fig. 1C). Namely, TrnC contains a canonical RiPP recognition element (RRE) – a winged helix-turn-helix structural motif that is found in a large number of RiPP biosynthetic enzymes and is typically involved in precursor peptide binding.^25^ This motif is composed of a 3-stranded β-sheet and a flanking 3-helical bundle. TrnD possesses a modified form of the RRE containing an intact 3-stranded β-sheet (the “wing”) but with a distorted helical bundle according the AF3 model (Fig. 1C). RiPP leader peptide binding to the β-sheet of the RRE has been validated by high-resolution structural biology in several different RiPP systems including the ranthipeptide, thermocellin, which is produced by a mechanistically similar rSAM enzyme (CteB).^2,26,27^

Surprisingly, the AF3 model suggests that the RREs of TrnC and TrnD interact extensively with each other to form the heterodimeric interface, suggesting a new role for the RRE in RiPP enzyme dimerization. This novel interaction between RREs was validated by deletion of the RREs coupled with binding studies, which resulted in a >40-fold decrease in the binding constant for TrnC/D heterodimerization. Moreover, despite the fact that both “wings” of the TrnC and TrnD RREs are exposed at the protein surface in the model, the TrnA and TrnB leader peptides are predicted by AF3 to interact preferentially with the RRE of TrnD (albeit with low confidence scores). While the structural model for the TrnA(B)/C/D complex requires further validation, this set of putative enzyme–enzyme/peptide interactions would not have been predicted based on our current understanding of RiPP–enzyme interactions. These findings suggest that functional protein–protein and protein–peptide interactions in RiPP biosynthesis may be vastly different across the RiPP family of natural products. Clearly, detailed mechanistic and structural studies are needed if researchers hope to gain a more complete understanding of the biophysical interactions of relevance to RiPP biosynthesis and engineering.

RiPP biosynthetic enzymes are charged with a daunting task – to install a precise set of chemical modifications into a dynamic RiPP precursor peptide while avoiding modification of the thousands of other ribosomally-produced polypeptides present in the cell. Understanding the molecular and physical basis of RiPP biosynthetic fidelity is critical for advancing RiPP engineering applications, but access to this knowledge is often hindered by the extreme variability in RiPP–enzyme intermolecular interactions and the reliance of the biosynthetic outcome on the dynamics of these interactions. As the community digs deeper into the molecular and structural logic of these intriguing RiPP systems, many more biosynthetic surprises are surely on the horizon.

## Author contributions

C. J. T. wrote the manuscript.

## Conflicts of interest

There are no conflicts to declare.
